# Supplementary material for: Compound- and context-dependent effects of antibiotics on greenhouse gas emissions from livestock
Source: R Soc Open Sci. 2019 Oct 30;6(10):182049. doi: 10.1098/rsos.182049 (PMC6837215; doi:10.1098/rsos.182049)
Supplement: Additional methods and results [file rsos182049supp1.docx]

Supplementary online material

Danielsson, R., Lucas, J., Dahlberg, J., Ramin, M., Agenäs, S., Bayat, A.R., Tapio, I., Hammer, T. & Roslin., T. 2019. Compound- and context-dependent effects of antibiotics on greenhouse gas emissions from livestock. *Royal Society Open Science*

# Rationale for compounds and doses used

To evaluate the effect of different antibiotic compounds on microbial community structure, and how it relates to the key functions of CH_4_ production and fiber digestibility, we focused on two of the antibiotics most commonly used to treat cattle in Sweden and Europe: penicillin and tetracycline (Swedish board of Agriculture 2016; De Briyne et al. 2014). Of these, tetracycline is a broad-spectrum antibiotic and acts against a wide range of gram positive and gram negative bacteria. Narrow-spectrum antibiotics such as benzyl penicillin mainly affect certain gram positive bacteria. To reflect realistic doses, we administered these compounds in modes and at doses typical of therapeutic treatment.

In Sweden, mastitis is the most common reason for treating dairy cows with antibiotics (Swedish Board of Agriculture 2012; De Briyne et al. 2014). Mastitis is an inflammation of the mammary gland, most often as a response to an intramammary infections. Intra mammary infection can be treated either locally by injecting the antibiotics through the teat canal into the affected udder quarter, or systemically by injection into muscle tissue or a blood vessel. In the latter cases, the antibiotic substance will be distributed to the entire body. In the case of local treatment of one udder quarter, antibiotics and antibiotic residues will reach the systemic circulation via blood circulating in the udder, but may not reach therapeutic concentrations outside the treated quarter. The main antibiotic used in treating mastitis in Sweden is benzyl penicillin (Swedish Board of Agriculture 2016). To reflect these practices and considerations, we chose the following treatments: in experiment 1 (Finland), the cows were treated with procaine benzylpenicillin, Penovet® vet. (suspension 300 mg/ml; Boehringer Ingelheim Vetmedical) 20 000 IU/kg injected intra muscularly once per day for 5 consecutive days. In Experiment 2 (Sweden), we used a 5-day treatment of procaine bensylpenicillin, Penovet® vet. (Boehringer Ingelheim Vetmedical) 20 mg/ kg BW intra muscularly once per day; a 5-day intra mammary treatment of Bensylpenicillinprokain, Carepen® vet. (Boehringer Ingelheim Vetmedical) 600 mg into the right front udder quarter and a 4-day treatment of tetracycline, Engemycin® vet. (Intervet) 10 mg/ kg BW given intra muscularly one time per day. All treatments were performed according to the instructions provided by the manufacturers.


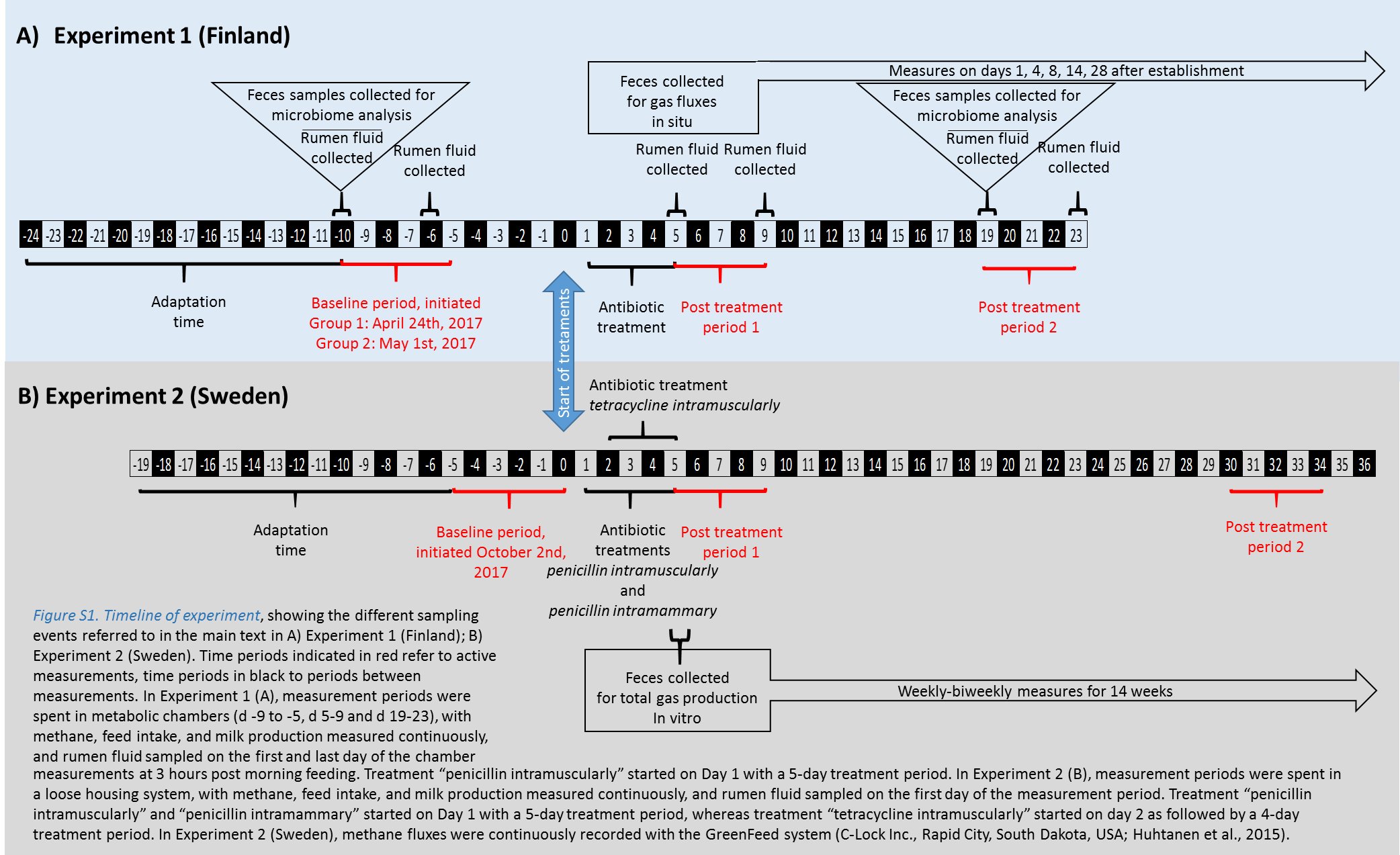


# Animals, feed and care during experiment

## Experiment 1 (Finland)

We used eight healthy Nordic Red cows from the Natural Resources Institute Finland (Luke) research barn. At the beginning of the trial, the animals had an average body weight of 614 kg (SD=50), days in milk of 418 (SD =42), milk yield of 19.5 kg milk per day (SD = 2.8) and somatic cell count of 94 300/mL (SD = 73 500).

The cows were fed diet consisting of concentrate ingredients and freely accessible grass silage (Timothy-Meadow fescue sward) ensiled with a formic acid-based additive (5 L/ton, AIV, Valio Ltd, Finland). Concentrates were calculated based on lactation stage and milk production of each cow and were available to cows from feeding kiosk and milking parlour. The composition of supplemental concentrate offered is presented in Table S1.1.

For the experiment, animals were randomly allocated to the control (n=4 animals) and antibiotic treatment (n=4 animals) in a completely randomized design. Due to the restricted number of respiration chambers (four in total), cows were split into two groups, with two treatment and two control cows in each. These groups of animals were fed and sampled with identical methodology, with an interval of one week. In the main paper, they are referred to as “Group 1” and “Group 2”.

During chamber measurement periods, cows were fed 4 times per day at 0730, 1300, 1700 and 1900 h. Inside chambers, the cows were milked at 0700 and 1645 h, respectively. Cows were restrained within the chamber by a yoke around the neck with access to a dedicated platform (L x W 1.80 x 1.26 m) covered with a rubber mat and continuous supply of fresh water and experimental feeds.

Representative samples of grass silage and concentrate were collected over chamber measurement days, kept at +4°C, composited for each period and stored at -20°C. Feed refusals were weighed daily during chamber measurements.

*Table S1.1.* *Composition of feed concentrate* fed to animals in Experiment 1 (Finland)

| Ingredient | Inclusion rate (g/kg DM) |
| --- | --- |
| Barley | 175 |
| Oat | 259 |
| Wheat | 133 |
| Molassed sugar beet pulp | 119 |
| Rapeseed meal | 286 |
| Mahti-Mira mineral^1^ | 28 |

^1^Vitamin and mineral premix (Mahti-Mira, Vilomix Finland Oy, Paimio, Finland) declared to contain: (g/kg) calcium, 220; magnesium, 64; sodium, 105; (mg/kg) zinc, 2.127; manganese, 1.025; copper, 1.18; iodine, 66; cobalt, 520; mineral selenium, 33; organic selenium, 37; (IU/g) vitamin A, 156; vitamin D_3_, 52; and dl-α tocopheryl acetate, 1.44.

## Experiment 2 (Sweden)

For experiment 2 (Fig. 1 in main paper), we used twenty-four healthy lactating Swedish Red dairy cows. At the start of the trial, these animals had an average weight of 575 kg (SD=85.2) at a mean of 157 days in milk (SD = 77.3) and yielding 24.6 kg milk per day (SD = 5.4). Cell count was below 150 000 cells/ml on composite milk for all cows in the two monthly milk recordings preceding the experiment. The cows were kept in an insulated loose-housing system and milked in a milking parlour twice a day, at 0600 and 1600 h

The cows were fed a total mixed ration (TMR) (grass silage/concentrate ratio 600/400 g/kg on a DM basis) ad libitum, with free access to drinking water. A stationary feed mixer (Nolan A/S, Viborg, Denmark) processed the rations, which were then delivered with automatic feeder wagons into feed troughs 4 times a day (0330, 0800, 1300, and 1730 h). The forage consisted of two silages (35:55 on DM basis) harvested from different primary growth grass swards, both dominated by timothy and red clover. The TMR also consisted of ensiled barley and heat treated rapeseed (ExPro). Concentrate given in the Green Feed station was a commercial concentrate mix (Komplett 180, Lantmannen, Malmö, Sweden; for nutrient contents see Table S1.2).

Individual feed intake was recorded daily throughout the trial in roughage intake control feeders (Insentec B. V., Marknesse, the Netherlands). Milk yield was recorded during all milkings with gravimetric milk recorders (SAC, S.A. Christensen and Co Ltd., Kolding, Denmark). The cows were weighed before the start of the trial and subsequently after morning milking. Silage, barley, ExPro and the concentrate feed were sampled twice in each period. All feed samples were oven-dried at 60°C for 48 h.

*Table S1.2.* *Composition of feed concentrate* (Komplett 180, Lantmannen, Malmö, Sweden) offered in the Green Feed station in Experiment 2 (Sweden).

| Nutrients per kg dry matter (DM) | Content |
| --- | --- |
| DM, % | 88 |
| Energy, MJ | 13.4 |
| Crude protein, g/kg DM | 180 |
| Crude fat, g/kg DM | 61 |
| Netral detergent fiber, g/kg DM | 225 |
| Starch, g/kg DM | 310 |
| Calcium, g/kgDM | 8.6 |
| Phosohorus, g/kg DM | 6.5 |
| Potassium, g/kg DM | 8.5 |
| Magnesium, g/kg DM | 4.5 |
|  |  |
| **Additives** |  |
| Vitamine A, IE/kg | 4000 |
| Vitamine D, IE/kg | 2000 |
| Vitamine E, mg/kg | 40 |
| Selenium mg/kg | 0.4 |
| Copper mg/kg | 10 |

# Quantification of gas emissions in vivo: methodological notes

To establish the effects of administration of different antibiotic compounds on gas emissions from enteric fermentation, we measured gas emissions in the two experiments by slightly different techniques. For a complete timeline of measurement periods, see Fig. S1. For a complete list of metrics derived, technique for measurement and meaning, see Table 1 of the main paper.

All instruments used in the experiments were carefully recalibrated, and the reliability and repeatability of measurements has consistently been high during our six years of experience using the GreenFeed system. Sensors that are used to measure concentrations of CH_4_ and CO2 uses the NDIR method. The NDIR method uses a light at one end of a tube and a sensor at the other end, and a light filter which only lets through light at a wavelength specifically absorbed by CH_4_. The light source does eventually burn out, but that is easily noticed since then the sensor instantaneously stops working. We have naturally performed the normal calibrations (for concentration and recovery), which will adjust for potential drift over time. The sensor monitoring the position of the head of the cow is also robust and accurate. The head sensor has to be cleaned from time to time, but when such a need arises, the GreenFeed system sends a notification per email, thus allowing us to take immediate action.

# Quantification of gas emissions from manure under field conditions: prevailing weather conditions


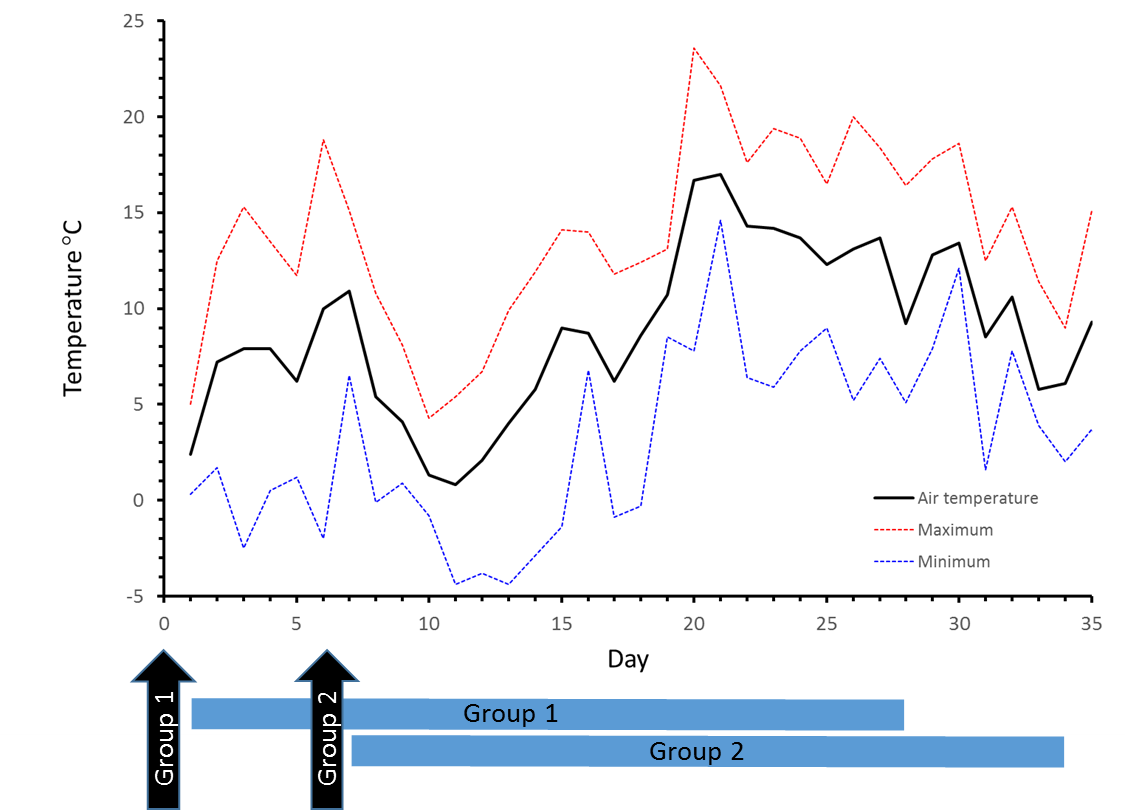


*Fig. S2. Temperature records* from the weather station closest to the experimental field in Viikki, Finland, during the measurements of gas emissions from dung under field conditions. Vertical arrows identify the dates of establishment of pats from Group 1 (April 30^th^) and Group 2 (May 6^th^), and blue bars their temporal extent. As can be seen, the first two weeks (including the start of Group 1) were particularly cold. Data from Finnish Meteorological Institute 2018; <http://en.ilmatieteenlaitos.fi/download-observations#!/>, accessed June 17, 2018.

# Additional results

## Antibiotic effects on multiple metrics

To achieve a comprehensive understanding of the effects of antibiotics on the digestive system of dairy cows, we targeted processes at all steps of feed use, from feed ingestion to fecal contents (see Table 1 of main paper for a categorization and complete tabulation of responses examined). Key patterns in terms of gas emissions and prokaryote community composition are reported in the main paper, whereas results on the other responses are reported below.

In all our analyses (see main paper), our main interest is in the main effect of treatment (identifying whether mean responses differed more between than within treatments) and in the interaction Treatment×Period, with periods being post-treatment period 1 and 2, identifying whether the recovery rate from a potential treatment effect varied with the treatment in question. Since we conduct multiple analyses of partly intercorrelated responses, we look for consistent patterns across individual responses rather than potentially spurious “significant” differences in terms of single response metrics.

Note that none of the responses were statistically significant (Table 1; Table S2–S5). To illustrate the levels of the metrics observed in our experiments, and the standard errors of the estimates (as an indicator of the power of the test), we show least squares means estimated of Treatment×Period for a few key metrics of each process addressed (see Table 1 of main paper).

### Metrics of Nutrient intake

Nutrient intake did not vary detectably among treatments in either Experiment 1 or 2 (Table S2). For each metric, the baseline consumption always emerged as significant, showing that a cow with a high intake originally would stay so throughout the experiments (Table S3). Differences between treatment were small and non-significant (Fig. S3).


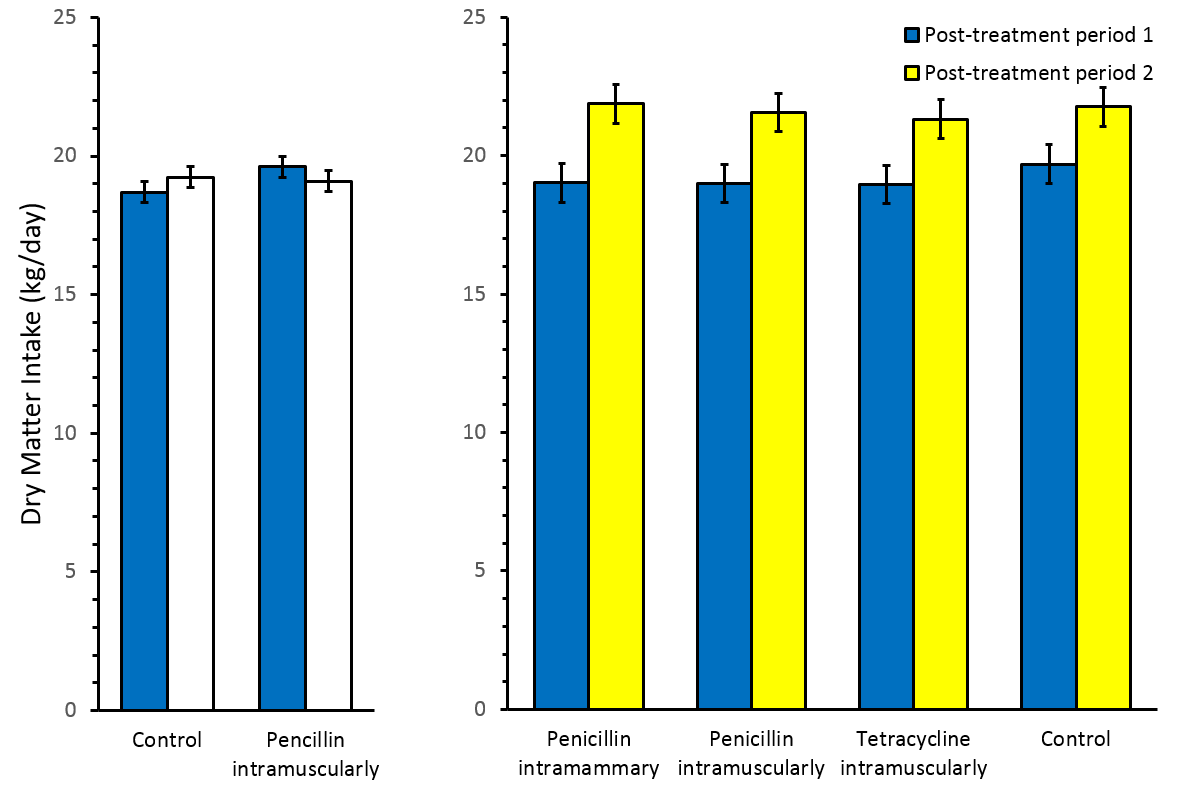


*Figure S3. Dry matter intake by cows in Experiments 1 and 2*. The figure shows least-squares means (±SE) from the GLMM described in Table S2, A-B. Blue bars refer to measurements taken a few days after antibiotic treatment (period 1), light bars to measurements taken 4 weeks after treatment (period 2; for a complete timeline of the respective experiments, see Fig. S1). To separate between results from Experiment 1 (Finland) and Experiment 2 (Sweden), panels are identified by the national colors.

*Table S2.* *Results from GLMM models of the nutrient intake of cows.* Shown are Type3-tests of fixed effects. Baseline refers to pre-treatment emissions, included as a covariate, whereas Group in Experiment 1 refers to two experimental cohorts of cows entering the metabolism chambers at different dates. Period refers to time since treatment (see Fig. S1 for a comprehensive timeline). Note that the baseline effect is always substantial, suggesting that cows characterized by initially high rates remain so throughout the experiment. To identify results from Experiment 1 (Finland) and Experiment 2 (Sweden), tables are separated by the national colors.

A) Dry matter (DM) intake, Experiment 1 (Finland)

| Effect | NDF | DDF | F | P |
| --- | --- | --- | --- | --- |
| Baseline | 1 | 10 | 26.20 | **0.0005** |
| Group | 1 | 10 | 0.97 | 0.3473 |
| Period | 1 | 10 | 0.00 | 0.9801 |
| Treatment | 1 | 10 | 1.05 | 0.3300 |
| Treatment×Period | 1 | 10 | 1.90 | 0.1984 |

B) Dry matter intake, Experiment 2 (Sweden)

| Effect | NDF | DDF | F | P |
| --- | --- | --- | --- | --- |
| Baseline | 1 | 19 | 22.82 | **0.0001** |
| Period | 1 | 20 | 51.78 | **<.0001** |
| Treatment | 3 | 19 | 0.18 | 0.9065 |
| Treatment×Period | 3 | 20 | 0.22 | 0.8779 |

C) Organic matter (OM) intake, Experiment 1 (Finland)

| Effect | NDF | DDF | F | P |
| --- | --- | --- | --- | --- |
| Baseline | 1 | 10 | 28.03 | **0.0004** |
| Group | 1 | 10 | 1.21 | 0.2972 |
| Period | 1 | 10 | 0.00 | 0.9824 |
| Treatment | 1 | 10 | 1.08 | 0.3230 |
| Treatment×Period | 1 | 10 | 1.92 | 0.1957 |

D) Crude protein intake, Experiment 1 (Finland)

| Effect | NDF | DDF | F | P |
| --- | --- | --- | --- | --- |
| Baseline | 1 | 10 | 36.59 | **0.0001** |
| Group | 1 | 10 | 1.86 | 0.2029 |
| Period | 1 | 10 | 0.29 | 0.6032 |
| Treatment | 1 | 10 | 0.90 | 0.3655 |
| Treatment×Period | 1 | 10 | 1.27 | 0.2866 |

E) Neutral detergent fiber (NDF) intake, Experiment 1 (Finland)

| Effect | NDF | DDF | F | P |
| --- | --- | --- | --- | --- |
| Baseline | 1 | 9.43 | 7.91 | **0.0194** |
| Group | 1 | 7.99 | 1.94 | 0.2008 |
| Period | 1 | 9 | 0.09 | 0.7715 |
| Treatment | 1 | 8.59 | 0.79 | 0.3983 |
| Treatment×Period | 1 | 9 | 1.79 | 0.2143 |

### Metrics of Rumen Fermentation

Indicators of rumen fermentation did not vary detectably among treatments in either Experiment 1 or 2 (Table S3). For many or most metrics, the baseline consumption emerged as significant, showing that a cow with a high initial value would stay so throughout the experiments (Table S3). Differences between treatments were small and non-significant (Fig. S4).


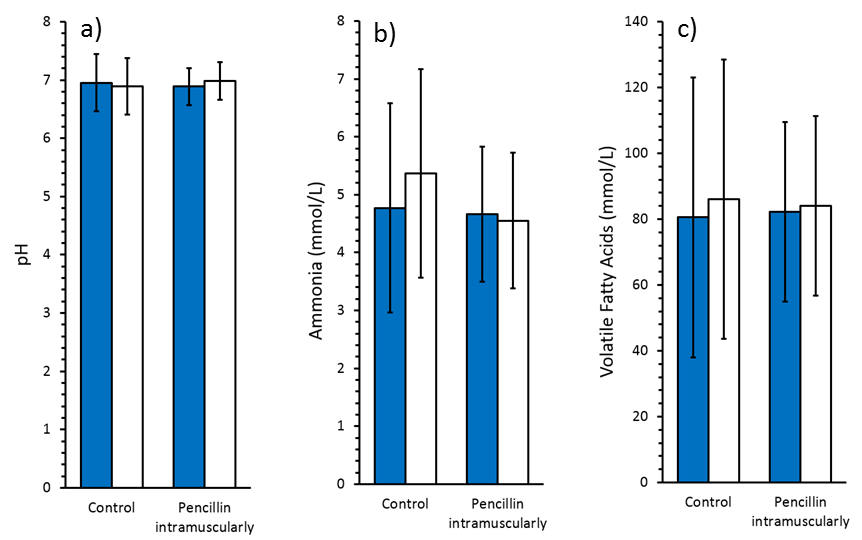


*Figure S4. A) Hydrogen ion pressure (pH), B) Ammonia concentration and C) Volatile fatty acids (VFA),* as observed in Experiment 1 (Finland). The figure shows least-squares means from the GLMM described in Table S3. Blue bars refer to measurements taken a few days after antibiotic treatment (period 1), light bars to measurements taken 4 weeks after treatment (period 2; for a complete timeline of the respective experiments, see Fig. S1).

*Table S3.* *Results from GLMM models of the nutrient intake of cows.* Shown are Type3-tests of fixed effects. Baseline refers to pre-treatment emissions, included as a covariate, whereas Group in Experiment 1 refers to two experimental cohorts of cows entering the metabolism chambers at different dates. Period refers to time since treatment (see Fig. S1 for a comprehensive timeline). Note that the baseline effect is always substantial, suggesting that cows characterized by initially high rates remain so throughout the experiment.

A) Ruminal hydrogen ion pressure (pH), Experiment 1 (Finland)

| Effect | NDF | DDF | F | P |
| --- | --- | --- | --- | --- |
| Baseline | 1 | 10 | 3.83 | 0.0789 |
| Group | 1 | 1 | 0.19 | 0.7396 |
| Period | 1 | 9 | 0.16 | 0.7013 |
| Treatment | 1 | 1.33 | 0.01 | 0.9498 |
| Treatment×Period | 1 | 9 | 2.95 | 0.1202 |

B) Ruminal ammonia concentration, Experiment 1 (Finland)

| Effect | NDF | DDF | F | P |
| --- | --- | --- | --- | --- |
| Baseline | 1 | 9.03 | 3.34 | 0.1007 |
| Group | 1 | 5.83 | 1.02 | 0.3523 |
| Period | 1 | 9 | 1.23 | 0.2965 |
| Treatment | 1 | 5.81 | 0.45 | 0.5268 |
| Treatment×Period | 1 | 9 | 2.59 | 0.1421 |

C) Ruminal volatile fatty acids (VFA), Experiment 1 (Finland)

| Effect | NDF | DDF | F | P |
| --- | --- | --- | --- | --- |
| Baseline | 1 | 9.71 | 11.72 | 0.0068 |
| Group | 1 | 1 | 0.00 | 0.9719 |
| Period | 1 | 9 | 0.85 | 0.3801 |
| Treatment | 1 | 1 | 0.00 | 0.9911 |
| Treatment×Period | 1 | 9 | 0.21 | 0.6540 |

D) Ruminal acetic acid, Experiment 1 (Finland)

| Effect | NDF | DDF | F | P |
| --- | --- | --- | --- | --- |
| Baseline | 1 | 10 | 5.57 | **0.0399** |
| Group | 1 | 10 | 4.98 | **0.0498** |
| Period | 1 | 10 | 1.56 | 0.2401 |
| Treatment | 1 | 10 | 0.00 | 0.9760 |
| Treatment×Period | 1 | 10 | 1.64 | 0.2291 |

E) Ruminal propionic acid, Experiment 1 (Finland)

| Effect | NDF | DDF | F | P |
| --- | --- | --- | --- | --- |
| Baseline | 1 | 10 | 23.18 | **0.0007** |
| Group | 1 | 10 | 5.92 | **0.0352** |
| Period | 1 | 10 | 0.77 | 0.4000 |
| Treatment | 1 | 10 | 0.64 | 0.4412 |
| Treatment×Period | 1 | 10 | 5.18 | **0.0461** |

F) Ruminal butyric acid, Experiment 1 (Finland)

| Effect | NDF | DDF | F | P |
| --- | --- | --- | --- | --- |
| Baseline | 1 | 9.05 | 62.81 | **<.0001** |
| Group | 1 | 10 | 1.67 | 0.2255 |
| Period | 1 | 9 | 0.00 | 0.9490 |
| Treatment | 1 | 9.99 | 4.78 | 0.0538 |
| Treatment×Period | 1 | 9 | 0.42 | 0.5313 |

G) Ruminal isobutyric acid, Experiment 1 (Finland)

| Effect | NDF | DDF | F | P |
| --- | --- | --- | --- | --- |
| Baseline | 1 | 9.96 | 1.92 | 0.1962 |
| Group | 1 | 1 | 0.03 | 0.8937 |
| Period | 1 | 9 | 1.03 | 0.3365 |
| Treatment | 1 | 1.05 | 0.31 | 0.6726 |
| Treatment×Period | 1 | 9 | 0.01 | 0.9291 |

H) Ruminal valeric acid, Experiment 1 (Finland)

| Effect | NDF | DDF | F | P |
| --- | --- | --- | --- | --- |
| Baseline | 1 | 10 | 2.53 | 0.1429 |
| Group | 1 | 10 | 3.31 | 0.0987 |
| Period | 1 | 10 | 9.11 | **0.0129** |
| Treatment | 1 | 10 | 0.01 | 0.9381 |
| Treatment×Period | 1 | 10 | 1.85 | 0.2034 |

I) Ruminal isovaleric acid, Experiment 1 (Finland)

| Effect | NDF | DDF | F | P |
| --- | --- | --- | --- | --- |
| Baseline | 1 | 9.02 | 0.58 | 0.4645 |
| Group | 1 | 9.77 | 2.20 | 0.1698 |
| Period | 1 | 9 | 6.07 | **0.0360** |
| Treatment | 1 | 9.93 | 2.55 | 0.1417 |
| Treatment×Period | 1 | 9 | 0.35 | 0.5708 |

J) Ruminal caproic acid, Experiment 1 (Finland)

| Effect | NDF | DDF | F | P |
| --- | --- | --- | --- | --- |
| Baseline | 1 | 10 | 0.05 | 0.8336 |
| Group | 1 | 10 | 0.00 | 0.9607 |
| Period | 1 | 10 | 3.56 | 0.0886 |
| Treatment | 1 | 10 | 0.36 | 0.5625 |
| Treatment×Period | 1 | 10 | 1.40 | 0.2635 |

K) Ruminal molar ratio of acetate to propionate, Experiment 1 (Finland)

| Effect | NDF | DDF | F | P |
| --- | --- | --- | --- | --- |
| Baseline | 1 | 10 | 10.71 | **0.0084** |
| Group | 1 | 10 | 3.44 | 0.0931 |
| Period | 1 | 10 | 1.13 | 0.3125 |
| Treatment | 1 | 10 | 0.61 | 0.4525 |
| Treatment×Period | 1 | 10 | 4.57 | 0.0582 |

### Metrics of Digestibility

Indicators of digestibility did not vary detectably among treatments in Experiment 1 (Table S4). For two out of four metrics, baseline values emerged as significant, showing that a cow with a high initial value would stay so throughout the experiments (Table S4). Differences between treatments were small and non-significant (Fig. S4).

*Table S4.* *Results from GLMM models of digestibility metrics of cows.* Shown are Type3-tests of fixed effects. Baseline refers to pre-treatment emissions, included as a covariate, whereas Group in Experiment 1 refers to two experimental cohorts of cows entering the metabolism chambers at different dates. Period refers to time since treatment (see Fig. S1 for a comprehensive timeline). Note that for two out of four metrics, the baseline effect is substantial, suggesting that cows characterized by initially high rates remain so throughout the experiment. All results derive from Experiment 1 (Finland).

A) Digestibility of organic matter (OM), Experiment 1 (Finland)

| Effect | NDF | DDF | F | P |
| --- | --- | --- | --- | --- |
| Baseline | 1 | 10 | 0.79 | 0.3937 |
| Group | 1 | 10 | 1.61 | 0.2336 |
| Period | 1 | 10 | 0.13 | 0.7234 |
| Treatment | 1 | 10 | 0.89 | 0.3678 |
| Treatment×Period | 1 | 10 | 0.99 | 0.3440 |

B) Digestibility of crude protein (CP), Experiment 1 (Finland)

| Effect | NDF | DDF | F | P |
| --- | --- | --- | --- | --- |
| Baseline | 1 | 10 | 5.52 | **0.0431** |
| Group | 1 | 10 | 0.03 | 0.8761 |
| Period | 1 | 10 | 0.16 | 0.6960 |
| Treatment | 1 | 10 | 0.38 | 0.6064 |
| Treatment×Period | 1 | 10 | 0.40 | 0.5445 |

C) Digestibility of neutral detergent fiber (NDF), Experiment 1 (Finland)

| Effect | NDF | DDF | F | P |
| --- | --- | --- | --- | --- |
| Baseline | 1 | 10 | 0.14 | 0.7131 |
| Group | 1 | 10 | 6.75 | **0.0270** |
| Period | 1 | 10 | 4.50 | 0.0628 |
| Treatment | 1 | 10 | 1.63 | 0.2305 |
| Treatment×Period | 1 | 10 | 1.35 | 0.2760 |

D) Digestibility of potentially-digestible NDF (pdNDF), Experiment 1 (Finland)

| Effect | NDF | DDF | F | P |
| --- | --- | --- | --- | --- |
| Baseline | 1 | 10 | 7.13 | **0.0235** |
| Group | 1 | 10 | 6.22 | **0.0318** |
| Period | 1 | 10 | 1.40 | 0.2640 |
| Treatment | 1 | 10 | 1.37 | 0.2693 |
| Treatment×Period | 1 | 10 | 0.05 | 0.8295 |

### Metrics of Milk Production

Milk production did not vary detectably among treatments in either Experiment 1 or 2 (Table S5). In both experiments, baseline production emerged as significant, showing that a cow with an originally high output of milk stayed highly productive throughout the experiments (Table S5). Differences between treatment were small and non-significant (Fig. S5).


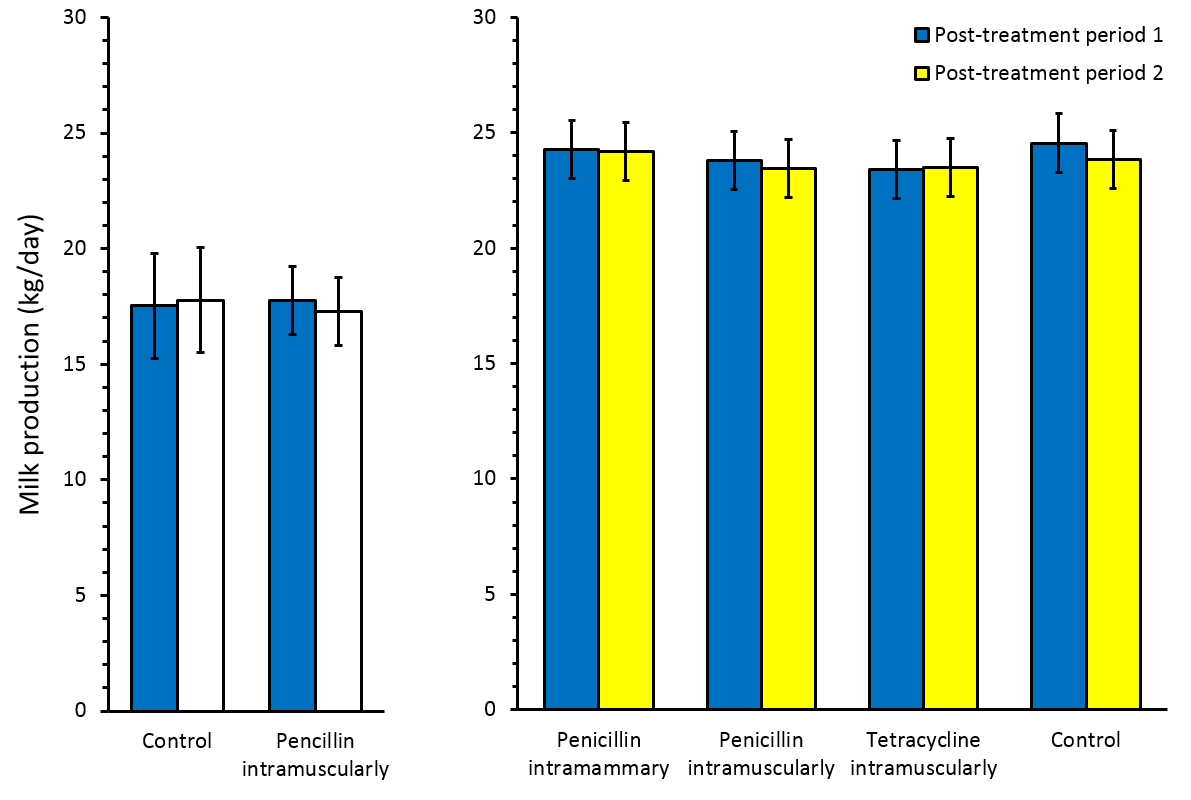


*Figure S5.* Milk production by the cows in Experiments 1 and 2. The figure shows least-squares means from the GLMM described in Table S5. Blue bars refer to measurements taken a few days after antibiotic treatment (period 1), light bars to measurements taken 4 weeks after treatment (period 2; for a complete timeline of the respective experiments, see Fig. S1). To separate between results from Experiment 1 (Finland) and Experiment 2 (Sweden), panels are identified by the national colors.

*Table S5.* *Results from GLMM models of milk production.* Shown are Type3-tests of fixed effects. Baseline refers to pre-treatment emissions, included as a covariate, whereas Group in Experiment 1 refers to two experimental cohorts of cows entering the metabolism chambers at different dates. Period refers to time since treatment (see Fig. S1 for a comprehensive timeline). Note that the baseline effect is always substantial, suggesting that cows characterized by initially high productivity remained so throughout the experiments. To identify results from Experiment 1 (Finland) and Experiment 2 (Sweden), tables are separated by the national colors.

A) Milk production, Experiment 1 (Finland)

| Effect | NDF | DDF | F | P |
| --- | --- | --- | --- | --- |
| Baseline | 1 | 29.6 | 175.94 | **<.0001** |
| Group | 1 | 2.82 | 0.35 | 0.5964 |
| Period | 1 | 72 | 0.28 | 0.5977 |
| Treatment | 1 | 4.04 | 0.03 | 0.8811 |
| Treatment×Period | 1 | 72 | 2.40 | 0.1257 |

B) Milk production, Experiment 2 (Sweden)

| Effect | NDF | DDF | F | P |
| --- | --- | --- | --- | --- |
| Baseline | 1 | 19 | 91.67 | **<.0001** |
| Period | 1 | 20 | 0.13 | 0.7182 |
| Treatment | 3 | 19 | 0.15 | 0.9315 |
| Treatment×Period | 3 | 20 | 0.06 | 0.9823 |

### Metrics of Gas Emissions from Enteric Fermentation

Total gas emissions measured from individual cows were highly consistent between measurements (Fig. S6), and did not detectably vary with treatment in either Experiment 1 or 2 (see main text for detailed results). Neither did any differences emerge when emissions were gauged by feed intake and milk output (Table S5; Fig. S7). Again, for each metric, the baseline emissions always emerged as significant, showing that a cow with high initial emissions stayed so throughout the experiments (Table S5).


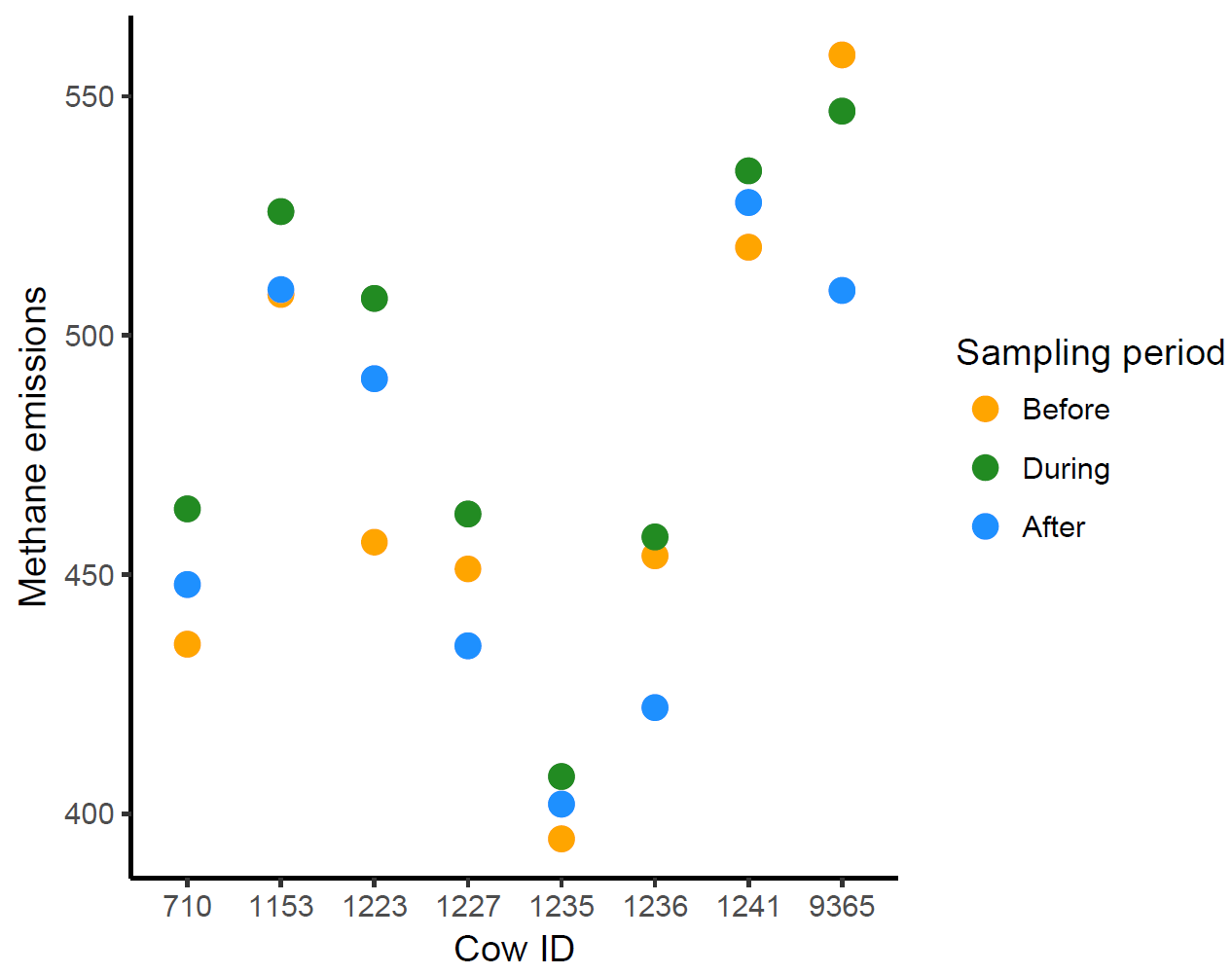


*Figure S6. A simple illustration of consistencies in methane emissions over time.* Here, we show the original data points on emissions from individual cows from the baseline period before the experiment versus during post-treatment period 1 and 2 in Experiment 1 (Finland). Note that individual cows produce highly similar levels of methane before, during and after antibiotic treatments.


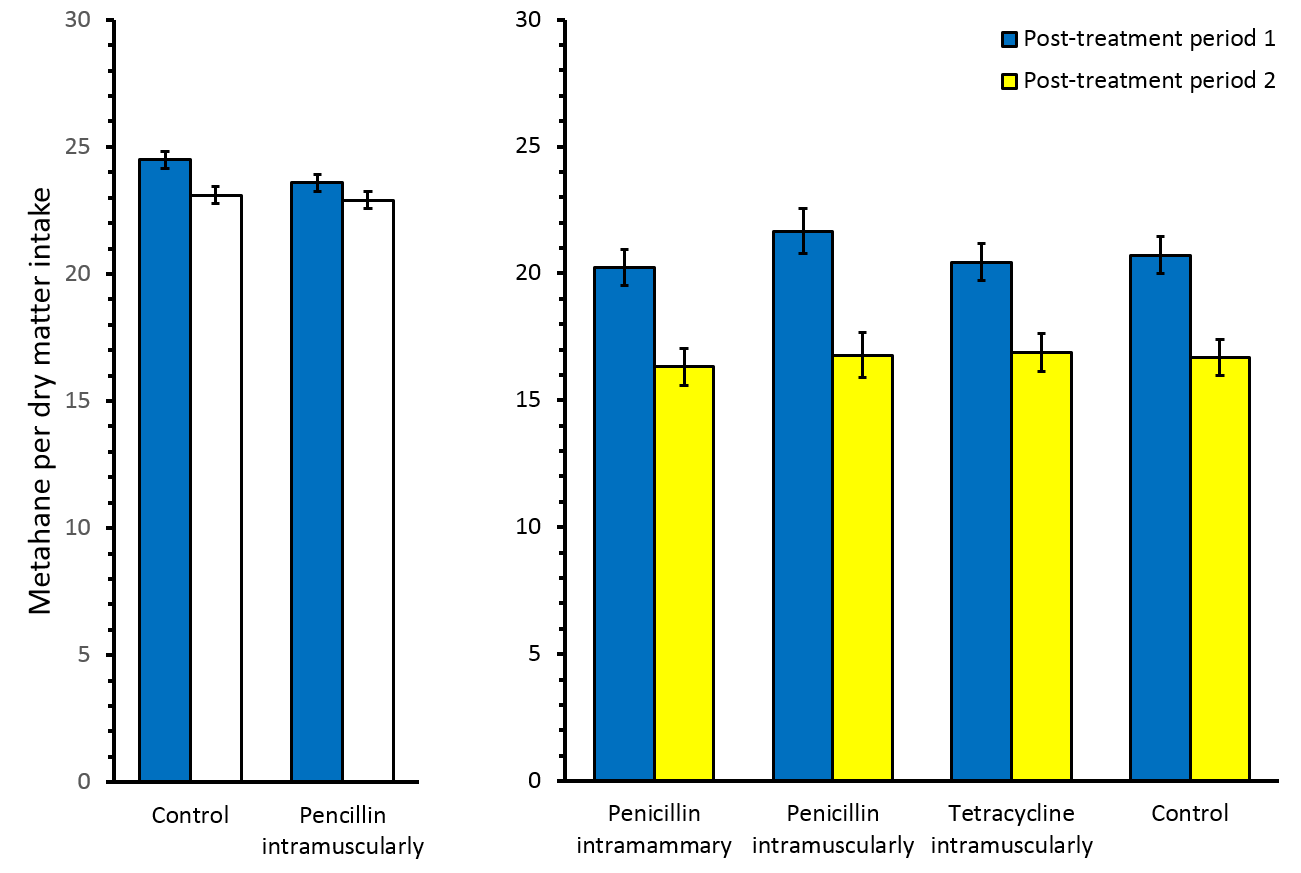


*Figure S7.* Methane yield scaled to dry matter intake, as observed in Experiments 1 and 2. The figure shows least-squares means from the GLMM described in Table S5. Blue bars refer to measurements taken a few days after antibiotic treatment (period 1), light bars to measurements taken 4 weeks after treatment (period 2; for a complete timeline of the respective experiments, see Supplementary Online Material). To identify results from Experiment 1 (Finland) and Experiment 2 (Sweden), panels are differentiated by national colors.

*Table S6.* *Results from GLMM models of gas emissions from enteric fermentation by cows.* Shown are Type3-tests of fixed effects. Baseline refers to pre-treatment emissions, included as a covariate, whereas Group in Experiment 1 refers to two experimental cohorts of cows entering the metabolism chambers at different dates. Period refers to time since treatment (see Fig. S1 for a comprehensive timeline). Note that the baseline effect is always substantial, suggesting that cows characterized by initially high rates remain so throughout the experiment. To identify results from Experiment 1 (Finland) and Experiment 2 (Sweden), tables are separated by the national colors.

A) Methane yield, Experiment 1 (Finland)

| Effect | NDF | DDF | F | P |
| --- | --- | --- | --- | --- |
| Baseline | 1 | 4 | 27.20 | **0.0064** |
| Group | 1 | 4 | 0.82 | 0.4154 |
| Period | 1 | 6 | 21.03 | **0.0037** |
| Treatment | 1 | 4 | 1.80 | 0.2504 |
| Treatment×Period | 1 | 6 | 2.49 | 0.1658 |

B) Methane yield, Experiment 2 (Sweden)

| Effect | NDF | DDF | F | P |
| --- | --- | --- | --- | --- |
| Baseline | 1 | 17 | 6.51 | **0.0206** |
| Period | 1 | 18 | 130.48 | **<.0001** |
| Treatment | 3 | 17 | 0.30 | 0.8273 |
| Treatment×Period | 3 | 18 | 0.53 | 0.6692 |

C) Methane emissions / unit OM intake, Experiment 1 (Finland)

| Effect | NDF | DDF | F | P |
| --- | --- | --- | --- | --- |
| Baseline | 1 | 4 | 28.35 | **0.0060** |
| Group | 1 | 4 | 0.99 | 0.3767 |
| Period | 1 | 6 | 21.76 | **0.0034** |
| Treatment | 1 | 4 | 1.81 | 0.2496 |
| Treatment×Period | 1 | 6 | 2.47 | 0.1668 |

D) Methane intensity, Experiment 1 (Finland)

| Effect | NDF | DDF | F | P |
| --- | --- | --- | --- | --- |
| Baseline | 1 | 4 | 15.01 | **0.0179** |
| Group | 1 | 4 | 1.40 | 0.3021 |
| Period | 1 | 6 | 4.12 | 0.0886 |
| Treatment | 1 | 4 | 0.39 | 0.5663 |
| Treatment×Period | 1 | 6 | 0.02 | 0.8836 |

E) Methane intensity, Experiment 2 (Sweden)

| Effect | NDF | DDF | F | P |
| --- | --- | --- | --- | --- |
| Baseline | 1 | 17 | 94.63 | **<.0001** |
| Period | 1 | 18 | 0.07 | 0.8009 |
| Treatment | 3 | 17 | 0.37 | 0.7771 |
| Treatment×Period | 3 | 18 | 0.20 | 0.8965 |

F) Total CO_2_, Experiment 1 (Finland)

| Effect | NDF | DDF | F | P |
| --- | --- | --- | --- | --- |
| Baseline | 1 | 4 | 35.32 | **0.0040** |
| Group | 1 | 4 | 5.12 | 0.0864 |
| Period | 1 | 6 | 14.33 | **0.0091** |
| Treatment | 1 | 4 | 0.11 | 0.7571 |
| Treatment×Period | 1 | 6 | 0.73 | 0.4251 |

G) CO_2_ yield, Experiment 1 (Finland)

| Effect | NDF | DDF | F | P |
| --- | --- | --- | --- | --- |
| Baseline | 1 | 4 | 20.50 | **0.0106** |
| Group | 1 | 4 | 0.88 | 0.4012 |
| Period | 1 | 6 | 16.58 | **0.0066** |
| Treatment | 1 | 4 | 0.60 | 0.4823 |
| Treatment×Period | 1 | 6 | 3.39 | 0.1151 |

H) CO_2_ emissions / unit OM intake, Experiment 1 (Finland)

| Effect | NDF | DDF | F | P |
| --- | --- | --- | --- | --- |
| Baseline | 1 | 4 | 22.30 | **0.0092** |
| Group | 1 | 4 | 1.01 | 0.3727 |
| Period | 1 | 6 | 16.92 | **0.0063** |
| Treatment | 1 | 4 | 0.60 | 0.4813 |
| Treatment×Period | 1 | 6 | 3.36 | 0.1167 |

I) CO_2_ intensity, Experiment 1 (Finland)

| Effect | NDF | DDF | F | P |
| --- | --- | --- | --- | --- |
| Baseline | 1 | 4 | 19.46 | **0.0116** |
| Group | 1 | 4 | 1.69 | 0.2630 |
| Period | 1 | 6 | 2.60 | 0.1582 |
| Treatment | 1 | 4 | 0.80 | 0.4229 |
| Treatment×Period | 1 | 6 | 0.10 | 0.7598 |

### Metrics of Prokaryote Community Structure

In Experiment 1 (Finland), cows in the two Groups in which treatments were initiated at different dates varied somewhat in their original prokaryotic community composition. Such differences were evident in terms of the microbial communities of the rumen and dung. For the rumen microbiota, differences between groups were evident during both post-treatment period 1 (Permanova pseudo-F_1,13_: 2.96, p = 0.001) and post-treatment period 2 (Permanova pseudo-F_1,13_: 2.01, p = 0.001), but not prior to treatment (Permanova pseudo-F_1,13_: 1.05, p = 0.47; Fig. S8). For the dung microbiota, no differences in the microbiomes among the two groups were evident before antibiotic treatment (Permanova pseudo-F_1,21_: 1.29, p = 0.12), whereas some differences among groups were evident after antibiotic treatment (Permanova pseudo-F_1,21_: 1.57, p = 0.01), thus generating a significant interaction between treatment and time (Permanova pseudo-F: 1.06, p = 0.001; Fig. S9).


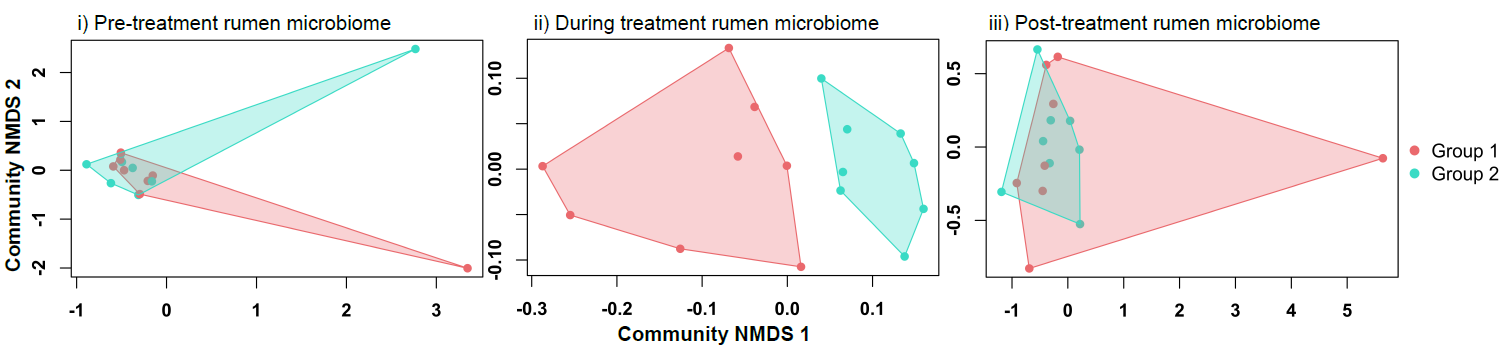


*Figure S8. Rumen microbial communities differ by group.* Figures represent prokaryotic community structure in Experiment 1 (Finland) i) before, ii) during and iii) after antibiotic treatment. These non-metric multidimensional scaling ordinations visually represent weighted Unifrac distances among samples in two dimensions. The two groups (as started on different days; Fig. S2) are identified by color, shape and corresponding polygon.


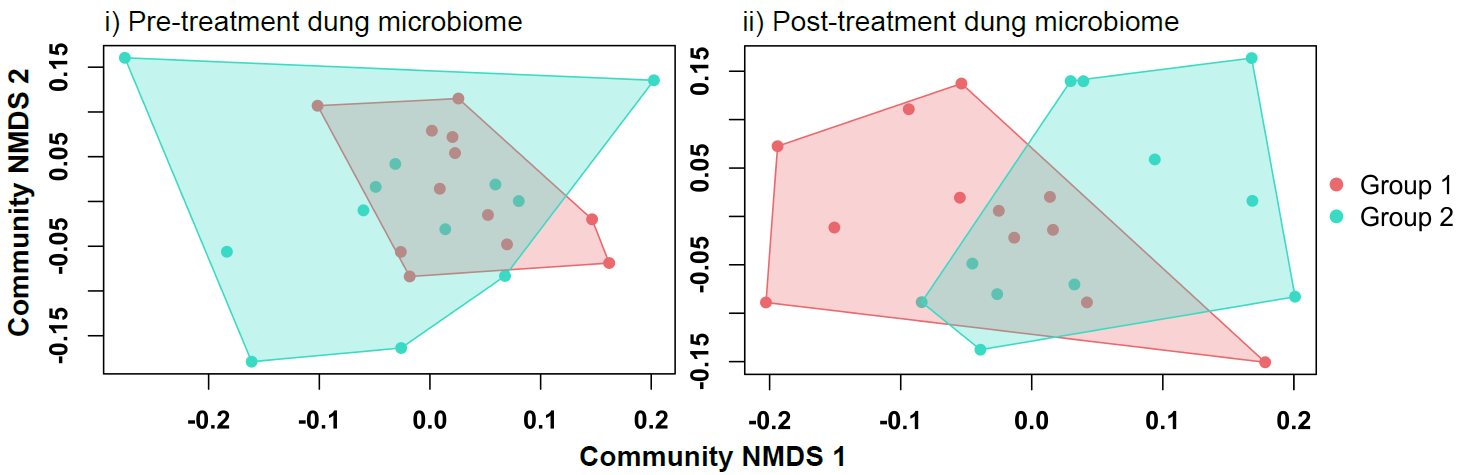


*Figure S9. Dung microbial communities differ by group.* Figures represent prokaryotic community structure (i) one week prior to antibiotic treatment and (ii) two weeks post antibiotic treatment. Shown are non-metric multidimensional scaling ordinations of microbiota in dung of cattle, representing weighted Unifrac distances among samples in two dimensions. The two groups (as started on different days; Fig. S2) are identified by color and corresponding polygon.

# References

De Briyne N, Atkinson J, Borriello SP, Pokludová L. 2014 Antibiotics used most commonly to treat animals in Europe. *Veterinary Record* **175**, 325–325. (doi:10.1136/vr.102462)

Swedish Board of Agriculture 2012. Official Statistics of Sweden. Animal Health 2012. See https://www.jordbruksverket.se/webdav/files/SJV/Amnesomraden/Statistik,%20fakta/Djurh%C3%A4lsa/JO25SM1201/JO25SM1301/JO25SM1301_inEnglish.htm. Accessed July 9, 2018.

Swedish board of Agriculture. 2016. Report. Dnr. 5.6.17-4368/16.
